# Bioflocculation potentials of a uronic acid-containing glycoprotein produced by *Bacillus* sp. AEMREG4 isolated from Tyhume River, South Africa

**DOI:** 10.1007/s13205-017-0695-8

**Published:** 2017-05-12

**Authors:** Nozipho Ntsangani, Kunle Okaiyeto, Nwodo U. Uchechukwu, Ademola O. Olaniran, Leonard V. Mabinya, Anthony I. Okoh

**Affiliations:** 1 SAMRC Microbial Water Quality Monitoring Centre, University of Fort Hare, Alice, 5700 South Africa; 2 Applied and Environmental Microbiology Research Group (AEMREG), Department of Biochemistry and Microbiology, University of Fort Hare, Alice, 5700 South Africa; 3 Department of Microbiology, School of Life Sciences, University of KwaZulu-Natal, Private Bag X54001, Durban, 4000 South Africa

**Keywords:** *Bacillus* sp. AEMREG4, Uronic acid-containing glycoprotein, Flocculating activity, Thermostable

## Abstract

Bioflocculants are secondary metabolites produced by microorganisms during their growth which have received attentions due to their biodegradability, innocuousness and lack of secondary pollution from degradation intermediates. This study reports on a bioflocculant produced by *Bacillus* specie isolated from Thyume River in South Africa. The bacterial isolate was identified through 16S rDNA sequencing and the BLAST analysis of the nucleotide sequences revealed 99% similarity to *Bacillus* sp. BCT-7112. The sequence was subsequently deposited in the GenBank as *Bacillus* sp. AEMREG4 with accession number KP406729. The optimum culture conditions for bioflocculant production were an inoculum size 4% (v/v) (80%) and starch (81%) as well as yeast extract (82%) as sole carbon and nitrogen sources, respectively. Addition of Ca^2+^ greatly enhanced the flocculating activity (76%) of crude bioflocculant over a wide range of pH 4–10 and retained high flocculating activity when heated at 100 °C for 1 h. Chemical analyses of the purified bioflocculant revealed carbohydrate (79% w/w) as a predominant component followed by uronic acid (15% w/w) and protein (5% w/w). Fourier transform infrared spectrum revealed the presence of carboxyl, hydroxyl and methoxyl groups as the functional groups responsible for flocculation and the high flocculation activity achieved portends its industrial applicability.

## Introduction

Flocculants are substances of either synthetic or natural origin used as sedimentation aids to bring about the solid–liquid separations by the process of flocculation (Lachhwani 2005). They are conventionally used in different industrial processes such as water treatment, downstream processing in fermentation processes and mineral ore treatment in metallurgical industries (Nwodo et al. 2013). Flocculants can either be cationic or anionic with respect to charge and they also exhibit a wide range of the molecular weights (Salehizadeh and Shojaosadati 2001). However, their setbacks include the hazard impose to human health such as neurotoxicity and carcinogenicity in humans (Dearfield et al. 1988). It has also been reported that aluminium, the main constituent of polyaluminum may lead to the development of Alzheimer’s disease (Arezoo 2002). Chemical flocculants such as polyacrylamides have also been reported to be recalcitrant to degradation (Mabinya et al. 2011; Piyo et al. 2011). Hence, the need for better alternatives that are safe and environmentally friendly and bioflocculants appear to be an important candidate to fill in the gap (Cosa et al. 2011).

Previous studies have confirmed the effectiveness of bioflocculants in the removal of humic acids, treatment of dye solutions, wastewater treatment and the removal of metal ions from aqueous solution (Salehizadeh and Shojaosadati 2003; Zouboulis et al. 2004; Deng et al. 2005; Liu et al. 2009). However, high production costs accompanied by low yield have been the major drawbacks to large-scale production for industrial applications; thus, prompting the need for new microorganisms with enhanced bioflocculant-producing capability that justify the production costs. In this paper, we report on the flocculating potential of a bioflocculant produced by *Bacillus* sp. AEMREG4 isolated from water samples from Tyhume River in the Eastern Cape Province, South Africa.

## Materials and methods

### Isolation and identification of bioflocculant-producing bacteria

The bacterial isolate was recovered from water samples collected from Tyhume River in the Eastern Cape Province, South Africa, screened for bioflocculant production and identified in accordance with our previous studies (Cosa and Okoh 2014; Nwodo et al. 2014; Ugbenyen and Okoh 2014). Briefly, seed culture of the bacterial isolate was prepared from the 20% v/v glycerol stock by inoculating 5 µl of the pure culture into 5 ml of the activation medium (Makapela et al. 2016), and incubated on a rotary shaker at 160 rpm, 28 °C for 24 h. The compositions of the growth medium contained the following: glucose 20 g, urea 0.5 g, yeast extract 0.5 g, (NH_4_)_2_SO_4_ 0.2 g, K_2_HPO_4_ 5 g, KH_2_PO_4_ 2 g, NaCl 0.1 g and MgSO_4_·7H_2_O 0.2 g in 1 l of distilled water (Nwodo et al. 2014). After 24 h of incubation, 2 ml of the seed culture was inoculated into 50 ml of growth medium and incubated on a rotary shaker at 160 rpm, 28 °C for 72 h. Thereafter, cell-free supernatant of the culture was obtained by centrifugation at 4000×*g*, 4 °C for 30 min and used to determine the flocculating activity.

### Determination of flocculating activity

A suspension of Kaolin (4 g/l) (Merck, Germany) was used as test material for estimating flocculating activity of the produced bioflocculant as described by Kurane et al. (1994). One hundred millilitres of clay suspension (100 ml), 3 ml of CaCl_2_ (1% w/v) and 2 ml of the culture supernatant were mixed together by agitation for 60 s in 250-ml conical flask and subsequently transferred into a 100-ml graduated cylinder, allowed to sediment for 5 min at room temperature. The control was also prepared similarly except for the bioflocculant which was replaced with a freshly prepared medium. The optical density (OD) of the clarifying supernatant was measured at 550 nm with a UV spectrophotometer (Thermo Spectronic, Heliox Epsilon, New York, USA) and the flocculating activity was calculated using the following formula:

$${\text{Flocculating activity }}(\% ) = \left( {{{{\text{A}} - {\text{B}}} \mathord{\left/ {\vphantom {{{\text{A}} - {\text{B}}} {\text{A}}}} \right. \kern-0pt} {\text{A}}}} \right) \times 100,$$

where A and B are optical densities of the control and sample, measured at 550 nm, respectively.

### Effect of inoculum size on bioflocculant production

The effect of inoculum size on bioflocculant production was assessed by varying the amounts of seed culture ranging from 1 to 5% (v/v) into a production medium as described by Ugbenyen et al. (2012). The cell-free supernatants were analysed for flocculating activity.

### Effect of carbon and nitrogen sources on bioflocculant production

The effect of various carbon and nitrogen sources on bioflocculant production was investigated in accordance with the method described by Ugbenyen et al. (2012). The seed culture was inoculated into growth medium contained in separate flasks supplemented with different carbon sources and incubated in a rotary shaker at 160 rpm, 28 °C for 120 h. The carbon source candidates included: glucose, fructose, maltose, lactose, sucrose and starch. Likewise, the nitrogen sources included: tryptone, peptone, urea, yeast extract (organic nitrogen sources) and ammonium sulphate (inorganic nitrogen source). The flocculating activity was determined after the cultivation period as described elsewhere.

### Effect of initial pH of growth medium

The effect of initial pH of the growth medium on bioflocculant production was examined by varying the pH of the production medium between the pH ranges of 4–10 using NaOH (0.1 M) or HCl (0.1 M) (Yim et al. 2007). The media were incubated in a rotary shaker at 160 rpm, 28 °C for 120 h after which flocculating activity was determined as previously described.

### Effect of cations on flocculating activity of crude bioflocculant

The effect of cations on the flocculating activity of crude bioflocculant was assessed following the same procedure as described elsewhere for determining flocculating activity; however, CaCl_2_ solution (1% w/v) used in the flocculating assay was replaced by various metal ion solutions prior to measuring flocculating activity. Solutions of NaCl, KCl (monovalent cations), MgCl_2_, MnCl_2_·4H_2_O, CaCl_2_ (divalent cations), AlCl_3_ and FeCl_3_·6H_2_O (trivalent cations) were used as cation sources (Liu et al. 2010) and flocculating activity was measured as previously described.

### Time course of bioflocculant production

#### Composition of culture medium

The optimum culture conditions for bioflocculant production were used for the time course assay as described by Okaiyeto et al. (2013). The medium compositions contained the following in 1 litre of distilled water: glucose 20 g, urea 0.5 g, yeast extract 0.5 g, (NH_4_)_2_SO_4_ 0.2 g, K_2_HPO_4_ 5 g, KH_2_PO_4_ 2 g, NaCl 0.1 g and MgSO_4_·7H_2_O 0.2 g. The pH was adjusted to pH 8 with either NaOH or HCl (0.1 M).

#### Standardization of inocula

For inoculum preparation, two loopful of the bacterial isolate from nutrient agar plate (Merck, South Africa) was inoculated into 50 mL of activation medium (beef extract 3 g, tryptone 10 g, NaCl 5 g in 1 litre of distilled water) in a 250-ml flask incubated on a rotary shaker at 160 rpm, 28 °C, for 24 h. After the cultivation period, distilled water was used to adjust the turbidity of the fermented broth to an optical density 0.1 (OD_600_) (Okaiyeto et al. 2015). The standardized bacterial suspension was inoculated into 200 ml of production medium and incubated in a rotary shaker at 160 rpm, 28 °C. Medium samples (15 ml) were withdrawn at 24-h intervals for a period of 120 h and monitored for cell growth, pH, and flocculating activity. Two millilitres of culture broth was centrifuged at 4000×*g*, 4 °C for 30 min, and the cell-free supernatant was used as the crude bioflocculant to determine the flocculating activity. The bacterial growth was monitored by measuring the viable counts using standard spread plate technique and nutrient agar and all plates were incubated at 37 °C for 36 h.

### Extraction and purification of bioflocculant

After 120 h of fermentation, the fermented broth was centrifuged at 4000×*g*, 4 °C for 30 min to remove cells and cell debris. The supernatant was diluted with 1 l of sterile distilled water and the mixture was centrifuged at 4000×*g*, 4 °C for 15 min to remove all the insoluble substances. Two volumes of cold ethanol were added to the supernatant and the mixture was stirred and left standing at 4 °C for 12 h for precipitation. The supernatant was discarded and the precipitate was vacuum-dried to obtain the crude biopolymer following the modified method of Zhang et al. (2010). The obtained precipitate was re-dissolved in distilled water (1% w/v) followed by the addition of one volume of a mixture of chloroform and n-butyl alcohol (5:2 v/v) with stirring. The mixture was then left standing at room temperature for 12 h and the upper phase was centrifuged at 4000×*g*, 4 °C for 15 min and thereafter, the supernatant was dialyzed overnight against distilled water. The dialysate was then vacuum-dried to obtain a purified bioflocculant.

### Characterization of the purified bioflocculant

The total protein content was measured by Folin–Lowry method as elaborated by Lowry et al. (1961) using bovine serum albumin (BSA) as the standard solution. The total sugar content of the purified bioflocculant was determined using the phenol–sulfuric acid method with glucose as the standard solution as described by Chaplin and Kennedy (1994). Potassium bromide (KBr) powder was ground with the dried bioflocculant before pressing it into pellets for Fourier transform infrared (FTIR) (Perkin Elmer System 2000, FT-IR, Middlesex, England) spectroscopy analysis over a wavelength range of 4000–370 cm^−1^ (He et al. 2010). The scanning electron microscopy (SEM) (JSM-6390LV, JEOL, Tokyo, Japan) imaging of the bioflocculant and the Kaolin clay particles was also examined (Xiong et al. 2010).

### Thermal stability of purified bioflocculant

Thermal stability of purified bioflocculant was carried out according to the method described by Okaiyeto et al. (2013). The bioflocculant solutions were incubated at various temperatures ranging from 50 to 100 °C for a period of 1 h prior to measuring residual flocculating activity at room temperature.

### Effect of pH and cations on the flocculating activity of purified bioflocculant

The effect of pH on the flocculating activity was determined by adjusting the pH of Kaolin clay suspension in a separate 250-ml flask and the pH values of the suspension ranging between 3 and 10 were adjusted using either HCl or NaOH (0.1 M) and thereafter, the flocculating activity of each suspension at different pH value was determined as previously described (Kurane et al. 1994). The effect of cations on the flocculating activity of purified bioflocculant was determined by replacing CaCl_2_ (1% w/v) with each of the following salt solutions: NaCl, KCl (monovalent cations), MgCl_2_, MnCl_2_·4H_2_O (divalent cations), AlCl_3_ and FeCl_3_·6H_2_O (trivalent cations) as cation sources (He et al. 2010; Liu et al. 2010). The flocculating activity was determined as previously described.

### Statistical analysis

All data were treated in triplicate and the mean values were taken. Data were subjected to one-way analysis of variance (ANOVA) using the MINITAB Student Release 12 statistical package for Windows 95/98 NT (Minitab Inc., State College, PA, USA, 2007).

## Results and discussion

### Isolation and identification of bioflocculant-producing bacteria

The test bacterium is Gram-positive with a circular-shaped cream coloured colonial morphology on nutrient agar. The 16S rDNA nucleotide sequence analyses of the bacterial isolate revealed it to have 99% similarity to *Bacillus* sp. BCT-7112. The nucleotide sequence was then deposited in GenBank as *Bacillus* sp. AEMREG4 with accession number KP406729. Quite number of *Bacillus* species have been reported to produce bioflocculants and these include *Bacillus mucilaginosus* (Deng et al. 2003), *Bacillus licheniformis* X14 (Li et al. 2009), *Bacillus* sp. Gilbert (Piyo et al. 2011), *Bacillus alvei* NRC-14 (Abdel-Aziz et al. 2011), and *Bacillus subtilis* MSBN17 (Sathiyanarayanan et al. 2013).

### Factors affecting bioflocculant production and flocculating activity

#### Effect of inoculum size on bioflocculant production

Inoculum size is a great significant factor in bioflocculant production and cell growth (Salehizadeh and Yan 2014). Salehizadeh and Shajoasadati (2001) reported that a small size of inoculum can prolong the lag phase, while the large inoculum size will create niches of the strain to overlap excessively; thus, inhibiting bioflocculant production. The effect of inoculum size on bioflocculant production in this study is shown in Table 1. An inoculum size of 4% (v/v) was optimal for bioflocculant production with flocculating activity of 80%, beyond which a decrease in activity was observed. Hence, 4% (v/v) inoculum size was used for the subsequent experiments. In corroboration with our findings, Xiong et al. (2010) documented a similar report for maximal bioflocculant production by *Bacillus licheniformis* at 4% (v/v) inoculum size. Also, in a study carried out by Okaiyeto et al. (2014) on a bioflocculant produced by *Micrococcus* sp. Leo, all the different inoculum sizes used resulted in flocculating activity of more than 80% with the maximum flocculating peak at 4% (v/v). On the contrary, studies carried out on the following bacterial strains, *Serratia ficaria* (Gong et al. 2008), *Klebsiella pneumoniae* YZ-6 (Luo et al. 2014) and by consortia of *Staphylococcus* sp. and *Pseudomonas* sp. (Zhang et al. 2007) for bioflocculant production suggest inoculum size of 1% as optimum. A bioflocculant production by *Aspergillus flavus* and *Virgibacillus* sp. Rob reached the highest flocculating activity at 2% (v/v) inoculum size (Aljuboori et al. 2013; Cosa et al. 2011).

**Table 1:** Effect of inoculum size on bioflocculant production by the test bacteria

Inoculum size (v/v %)	1	2	3	4	5
FA	77.55	75.03	76.98	79.38	48.30
(%) ±SD	1.63	1.80	1.73	0.45	2.14

The results are represented as mean value of triplicates ±SD

*FA* flocculating activity, *SD* standard deviation

#### Effect of carbon source on bioflocculant production

**Fig. 1:**
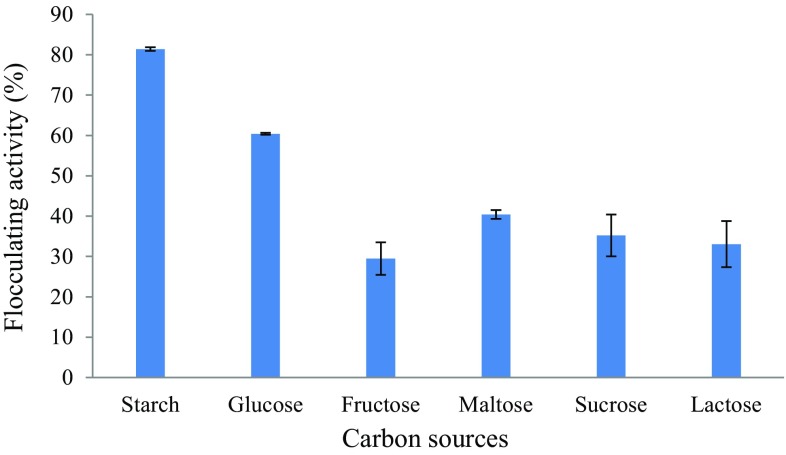
Effect of carbon sources on bioflocculant production by the test bacteria

Carbon source plays an important role in enhancing bioflocculant production and different microorganisms prefer different carbon sources for bioflocculant production (Piyo et al. 2011). The effect of various carbon sources on bioflocculant production in this study is presented in Fig. 1. Among the different carbon sources studied, starch was the most favourable carbon source favouring bioflocculant production with a flocculating activity of 81% and maltose being the least favourable carbon source with flocculating activity of 29%. Starch was then chosen as the sole carbon source for all the subsequent experiments. Similar findings were reported by Li et al. (2009) on bioflocculant production by *Bacillus licheniformis* X14, in which starch, sucrose and ethanol were the favourable carbon sources. Also, Deng et al. (2005) documented similar findings for *Aspergillus parasiticus* in which 96% flocculating efficiency was achieved with starch as a sole carbon source. Contrary to these findings, however, starch showed the least flocculating activity of 5% for *Virgibacillus* sp. Rob (Cosa et al. 2011) and completely inhibited bioflocculant production by *Halomonas* sp. OKOH (Mabinya et al. 2011). These findings support the observations that preferences for carbon source vary among bioflocculant-producing microorganisms (Salehizadeh and Yan 2014).

#### Effect of nitrogen sources on bioflocculant production

**Fig. 2:**
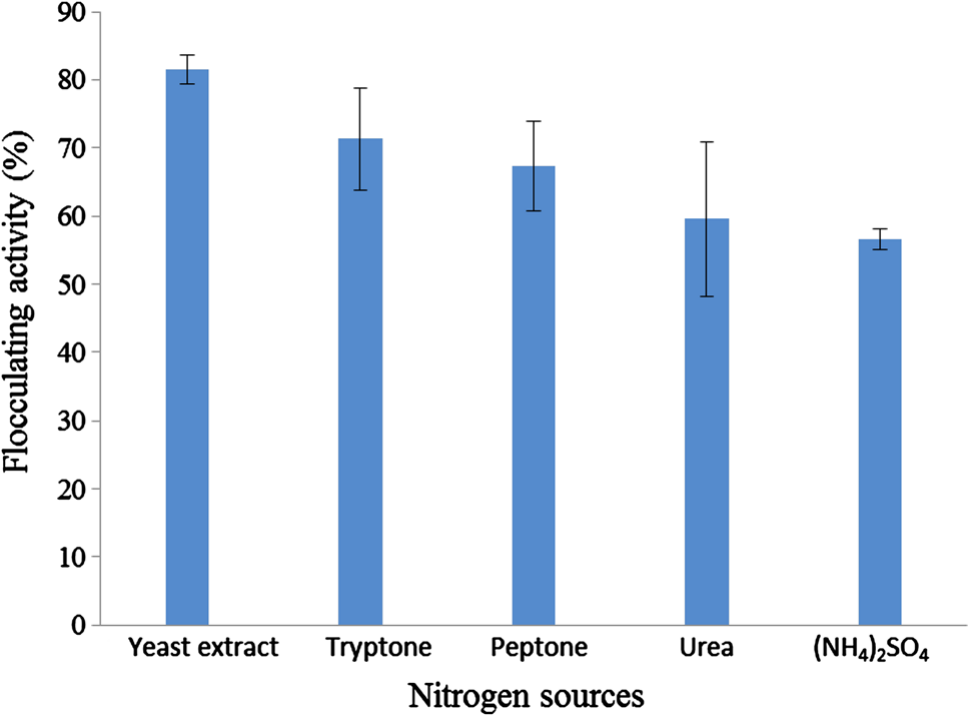
Effect of nitrogen sources on bioflocculant production by the test bacteria

Various microorganisms require the presence of either organic or inorganic nitrogen sources for bioflocculant production (Ugbenyen et al. 2012). The effect of organic (yeast extract, tryptone, peptone, urea) and inorganic nitrogen sources (ammonium sulphate) on bioflocculant production by *Bacillus* sp. was investigated. As shown in Fig. 2, among the various nitrogen sources examined, yeast extract proved to be the best nitrogen source with the highest flocculating activity of 82% and (NH_4_)_2_SO_4_ was the least favourable nitrogen source with a flocculating activity of 57%. Similarly, the presence of yeast extract as sole nitrogen source showed maximal flocculating activity on a bioflocculant produced by *Penicillium* sp. (Liu et al. 2010). In another study carried out by Zheng et al. (2008), the production of bioflocculant by *Bacillus* sp. F19 showed maximal flocculating activity of 78% when yeast extract was used as a sole nitrogen source. Shadia et al. (2011) investigated the effect of various nitrogen sources on bioflocculant production by *Bacillus alvei* NRC-14 and reported both (NH_4_)_2_SO_4_ and yeast extract to be the most effective nitrogen sources. It has been previously reported that organic nitrogen sources are easily absorbed by the cells; hence, they are most favourable for bioflocculant production when compared to inorganic nitrogen sources (Cosa et al. 2013).

#### Effect of the initial pH of growth medium on bioflocculant production

**Fig. 3:**
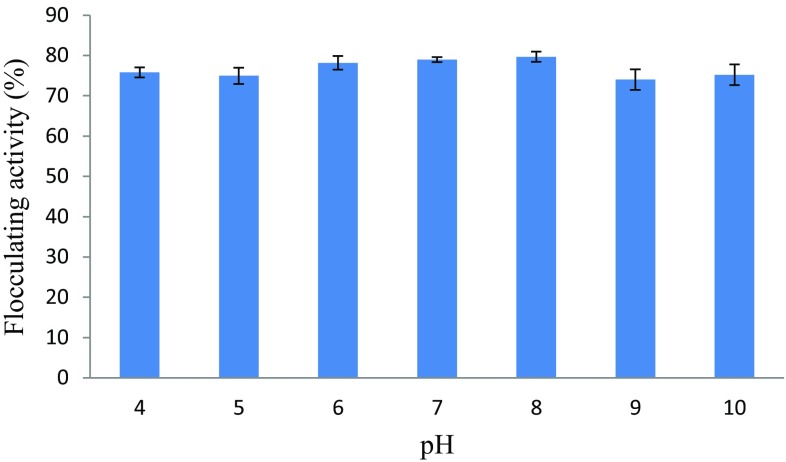
Effect of initial pH on bioflocculant production by the test bacteria

**Fig. 4:**
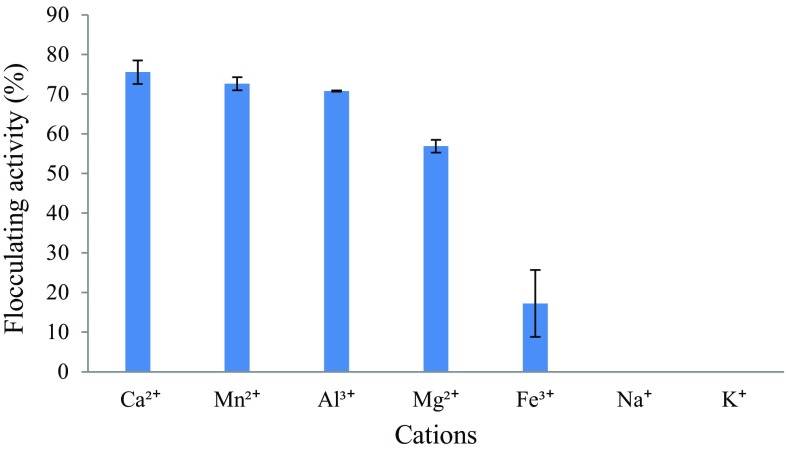
Effect of cations on the flocculating activity of the bioflocculant

Initial pH of growth medium for bioflocculant production varies with different microorganisms (Salehizadeh and Yan 2014). The effect of initial pH of the growth medium on bioflocculant production was investigated at a pH range of 4–10 and the results are depicted in Fig. 3. In this study, bioflocculant production by *Bacillus* sp. remained relatively constant at a wide range of pH 4–10 with flocculating activity between 74 and 80% as shown in Fig. 4. Optimum flocculating activity was reached at pH 8 (80%). According to the findings of He et al. (2010), the flocculating activity of a bioflocculant produced by *Halomonas* sp. V3a’ was above 80% at the pH range of 3–11 and the highest flocculating activity of 97% was recorded at pH 7. The optimum initial pH of growth medium for the production of bioflocculants by *Bacillus* xn12 and *Streptomyces* xn17 strains was in the range of 3–10 and 3–9, respectively, with the highest flocculating activity (97%) being observed at pH 5 for both strains (Zhang et al. 2013). Bioflocculant production by *Aspergillus flavus* was investigated at a pH range of 5–9 and the highest flocculating activity was reached at pH 7 (Aljuboori et al. 2013), whereas Zheng et al. (2008) noted maximum flocculating activity at pH 9 when culturing *Bacillus megaterium* within a wide pH range ranging from acidic to an alkaline pH of 12. Contrary to the above observations, *Anabaera* sp. produced a bioflocculant utilizing a very acidic medium of pH 2 (Choi et al. 1988).

#### Effect of cations on the flocculating activity of crude bioflocculant

Addition of cations enhance flocculating efficiency by charge neutralization and by forming bridges between particles (Salehizadeh and Shojaosadati 2001; Salehizadeh and Yan 2014). Bioflocculant efficiency is stimulated not only by the presence and concentration of cations but is also greatly influenced by the valence of ions (Zulkeflee et al. 2012). The effects of various cations on the flocculating activity of bioflocculant produced by *Bacillus* sp. AEMREG4 were examined and the results are illustrated in Fig. 4. The effect of cations with various valence ions (monovalent, divalent and trivalent cations) on flocculating activity was studied. Amongst the cations tested, Ca^2+^ enhanced flocculating rate with the highest activity of 76%, followed by Mn^2+^, Al^3+^ and Mg^2+^ at 73, 71 and 57%, respectively with observed significant inhibition by Fe^3+^ (17%) and complete inhibition by Na^+^ and K^+^. Similar results were observed in a study conducted by Mabinya et al. (2011), whereby the flocculating activity of a bioflocculant produced by *Halomonas* sp. OKOH was increased by the addition of divalent cations with Ca^2+^ being the most effective. Ugbenyen and Okoh (2014) also reported that the divalent cations tested (Ca^2+^, Mg^2+^, Mn^2+^) were the best metal ions that enhanced the activity of the bioflocculant produced by the consortium of the *Cobetia* and *Bacillus* species which in turn was completely inhibited by Li^+^ and K^+^. In this study, monovalent cations (Na^+^ and K^+^) had no effect at all on flocculating activity which can be attributed to the loose bonds in its structure and consequently resulted in a decrease in floc density, size and floc resistance to shear as compared to divalent cations (Zulkeflee et al. 2012).

#### Time course of bioflocculant production

**Fig. 5:**
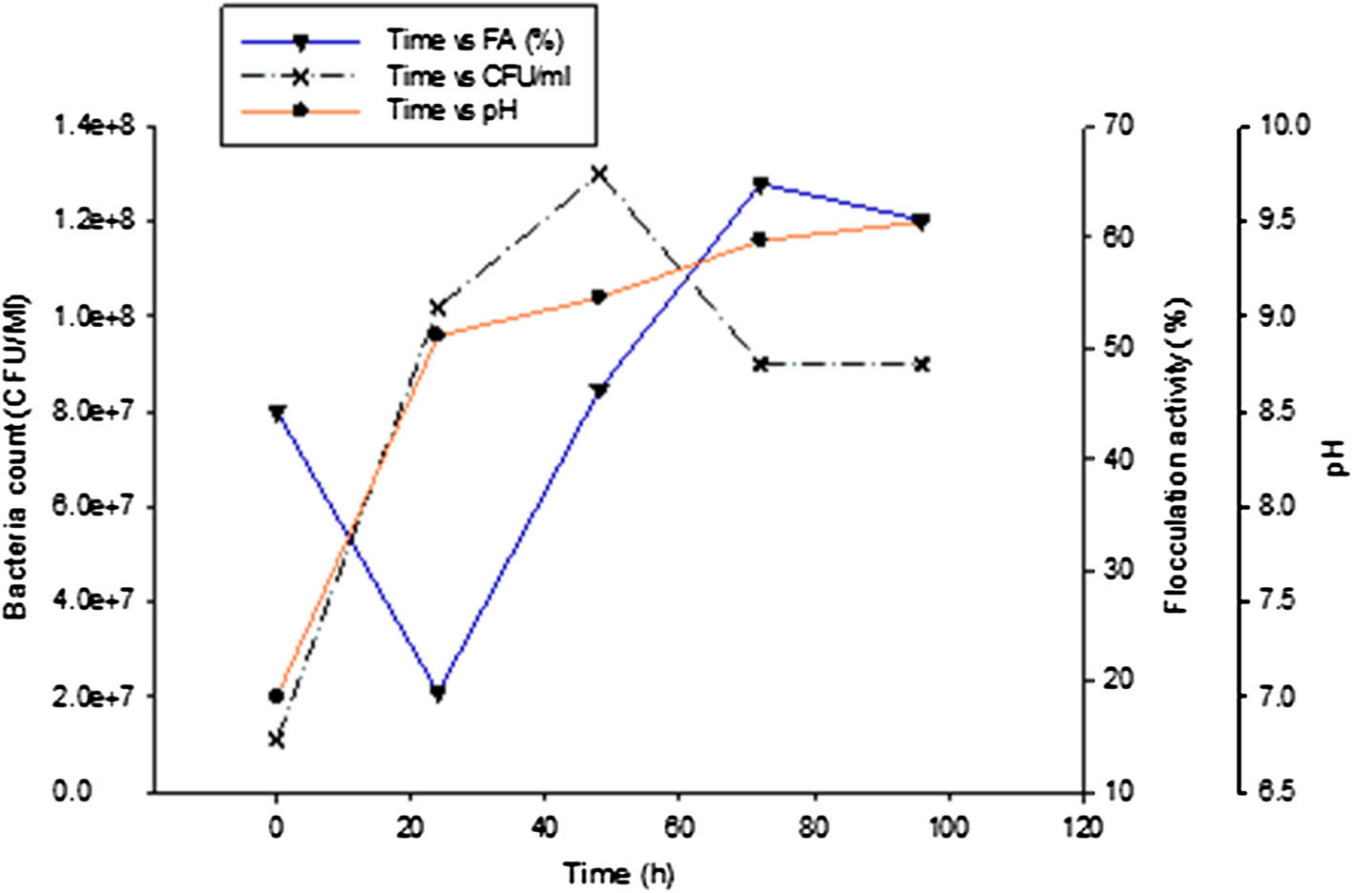
Time course of bioflocculant production by *Bacillus* sp.

Pre-determined optimal culture conditions were used for the time course assay and Fig. 5 depicts the time course assay of bioflocculant production by *Bacillus* sp. over a cultivation period of 96 h. Bioflocculants can be distinguished into either primary or secondary metabolites depending on the period of secretion in the culture broth (Salehizadeh and Yan 2014). The seed culture of the studied bacterial isolate prepared after 24 h of incubation was used for inoculation and since the bacteria is a bioflocculant-producing microbe, the small amount of the bioflocculant produced during this seed culture preparation was also utilized by the bacteria (used as a source of carbon) for growth and hence, a reduction in flocculating activity was observed at 24 h. Subsequently, as the bacteria utilized the nutrients in the basal medium, an increase in the flocculating activity of the bioflocculant was observed and optimal production was observed at 72 h with flocculating activity (65%) and beyond this cultivation period, a decline in the flocculating activity was recorded. A decline in cell growth and flocculating activity at late growth phase may be attributed to the depletion of nutrients in the production medium as well as to the production and release of bioflocculant-degrading enzymes which utilize the produced bioflocculant as a carbon source (Li et al. 2009; Zaki et al. 2011). Similarly, a bioflocculant produced by *Serratia ficaria* reached its maximum flocculating activity in the early stationary phase (72 h) and subsequently observed a slow decrease after 84 h which was attributed to autolysis and enzymatic activity (Gong et al. 2008). It was also observed that at the early stationary phase, there was a rapid increase in bacterial cell growth up to 48 h of incubation, as expressed in CFU/ml, after which a significant decrease in viable cells was observed eventually levelling off at 72 h with an observed gradual decrease in flocculating activity (Fig. 5). These findings indicated that the production of the bioflocculant is associated with cell growth rather than with cell autolysis (Lu et al. 2005; Gao et al. 2006). The majority of reported studies in the literature revealed that bioflocculants are produced during active growth phase of microorganisms (Prasertsan et al. 2006; Lian et al. 2008; Nwodo et al. 2013; Salehizadeh and Yan 2014). The present study seems to show a similar phenomenon. On the contrary, the bioflocculant produced by *Chryseobacterium daeguense* W6 was associated with cell autolysis and not cell growth (Liu et al. 2010). Running parallel to the profile of flocculating activity, the pH profile showed that the pH increased from 7.0 to 9.5 within an incubation period of 96 h (Fig. 5). Bioflocculant production takes place at various phases of growth of microorganisms. At various periods, production drops due to cell autolysis, metal complexing or decreased enzymatic activity depending, nevertheless, on the microbial cell culture (Lu et al. 2005; Vatansever, 2005; Cosa, 2010). A bioflocculant produced by *Streptomyces* sp. reached its highest flocculating activity during the logarithmic growth phase, suggesting that biosynthetic processes were responsible for the bioflocculant production process (Nwodo et al. 2012). Shih et al. (2001) reported that both cell growth and bioflocculant production by *Bacillus licheniformis* simultaneously reached the highest peak during the stationary phase at 96 h. Based on the report of Cosa et al. (2011), a bioflocculant produced by *Virgibacillus* sp. Rob was reported to produce a bioflocculant with maximum flocculating activity reached at 96 h of cultivation, whereas Okaiyeto et al. (2016) documented that the bioflocculant produced by *Bacillus* sp. reached its maximum flocculating activity of 94.9% at pH 6.17 attained after 72 h.

#### Chemical compositions analyses of the purified bioflocculant

From Table 2, chemical analyses of the purified bioflocculant revealed it to be composed of total sugar (79% w/w) and total protein (5% w/w), suggesting that the bioflocculant is predominantly polysaccharide. Further analysis showed that the bioflocculant consisted of uronic acid (15% w/w), which is an acidic polysaccharide. Several bioflocculants have been reported to have polysaccharide backbone as a major component, for example, the bioflocculant produced by *Enterobacter aerogenes* (Lu et al. 2005), the bioflocculant produced by *Virgibacillus* sp. Rob (Cosa et al. 2011), and the bioflocculant MBF-5 produced by *Klebsiela pneumonia* (Zhao et al. 2013).

**Table 2:** Chemical compositions of the purified bioflocculant produced by *Bacillus* sp.

Component	Percentage (%)
Total protein	5
Total sugar	79
Uronic acid	15

#### Functional groups analyses of purified bioflocculant

**Fig. 6:**
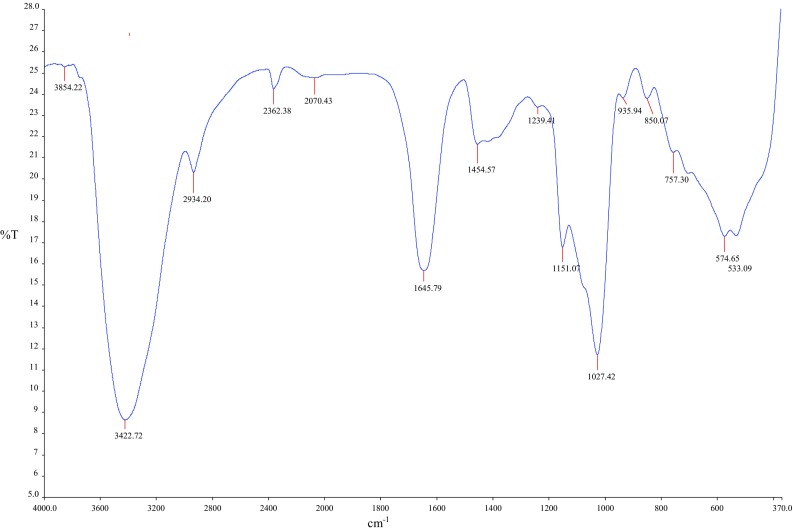
Infrared spectrum of the purified bioflocculant produced by *Bacillus* sp.

FTIR analysis of the bioflocculant was undertaken to detect the presence of any functional groups that may contribute to its flocculating activity as shown in Fig. 6. The spectrum displayed a broad stretching intense peak at 3423 cm^−1^ which is a characteristic of hydroxyl group. A weak C–H stretching vibration band was observed at 2934 cm^−1^. The bands at 1646 and 1455 cm^−1^ are characteristic of C=O asymmetrical and weak symmetrical stretching in the carboxylate, respectively (Deng et al. 2003), which represent the presence of carboxyl group in the structure of the bioflocculant produced by *Bacillus* sp. The bands at 1027 and 1151 cm^−1^ represent the methoxyl group (Zheng et al. 2008). The sorption peak at 1239 cm^−1^ indicates the presence of C–O stretching in ether or alcohol (Desouky et al. 2008). The absorption peaks around 1000–1100 cm^−1^ are known to be characteristic of all sugar derivatives (Zheng et al. 2008). The FTIR spectra showed the presence of carboxyl, hydroxyl and methoxyl groups, which are the preferred groups for flocculation (Zheng et al. 2008).

#### Scanning electron microscopy (SEM) images of bioflocculant

**Fig. 7:**
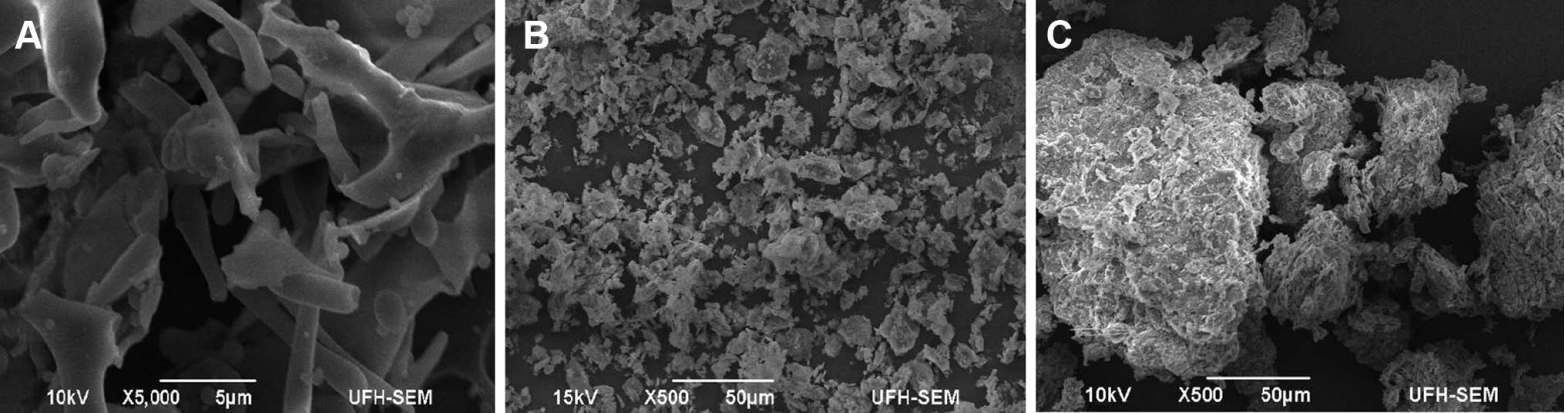
SEM images of **a** the purified bioflocculant, **b** Kaolin clay particles, and **c** Kaolin clay flocculated by purified bioflocculant produced by *Bacillus* sp.

SEM observations were carried out to study the morphological structures of the bioflocculant before and after flocculation. Figure 7a shows a rod-shaped structure of the purified bioflocculant. Figure 7b shows Kaolin clay particles before the addition of bioflocculant and Fig. 7c shows flocculated Kaolin clay after the addition of the purified bioflocculant. Comparing b and c, it can be deduced that the addition of the purified bioflocculant produced by the test bacteria to Kaolin clay suspension played a crucial role in connecting the scattered Kaolin particles together to form flocs and separate easily from water during the flocculation process.

### Factors influencing the flocculating activity of the purified bioflocculant

#### Effect of cations on the flocculating activity of the purified bioflocculant

The effect of cations on the flocculating activity of the purified bioflocculant was investigated as shown in Table 3. All tested cations enhanced the flocculating activity of the bioflocculant produced by *Bacillus* sp. AEMREG4 albeit to varying degrees: Ca^2+^ (78%), Mn^2+^ (77%), Mg^2+^ (70%), Al^3+^ (80%) and K^+^ (60%) with Na^+^ (47%) and Fe^3+^ (48%) showing the least effect (Table 3). The main cause of the poor flocculation of the bioflocculant produced by *Bacillus* sp. AEMREG4 in the presence of Fe^3+^ compared to Al^3+^ may be due to the synergistic effect of Al^3+^ with this bioflocculant and the antagonistic effect of Fe^3+^ with the bioflocculant; hence, resulting in high flocculating activity of Al^3+^ and lower flocculating activity of Fe^3+^ (Okaiyeto et al. 2015). Divalent and trivalent cations are said to have the effect of stimulating the adsorption of bioflocculant on the suspended particles by decreasing the negative charge of both the polymer and the particle (Levy et al. 1992). The bioflocculant produced by a consortium of *Holomonas* sp. Okoh and *Micrococcus* sp. Leo showed the maximum flocculating activity of 80% when Al^3+^ was the cation of choice (Okaiyeto et al. 2013). Stimulation of flocculating activity was observed in a bioflocculant produced by *Bacillus licheniformis* and *Bacillus circulans* when Al^3+^ and Ca^2+^ were used (Li et al. 2009).

**Table 3:** Effect of cations on the flocculating activity of the purified bioflocculant

Cation	Ca^2+^	Mn^2+^	Mg^2+^	Al^3+^	Fe^3+^	Na^+^	K^+^
FA (%)	77.81	76.96	70.41	79.99	47.96	47.18	59.47
±SD	3.57	6.39	2.22	3.59	1.56	5.16	7.40

The results are represented as mean value of triplicates ±SD

*FA* flocculating activity, *SD* standard deviation

#### Thermal stability test of the purified bioflocculant

**Fig. 8:**
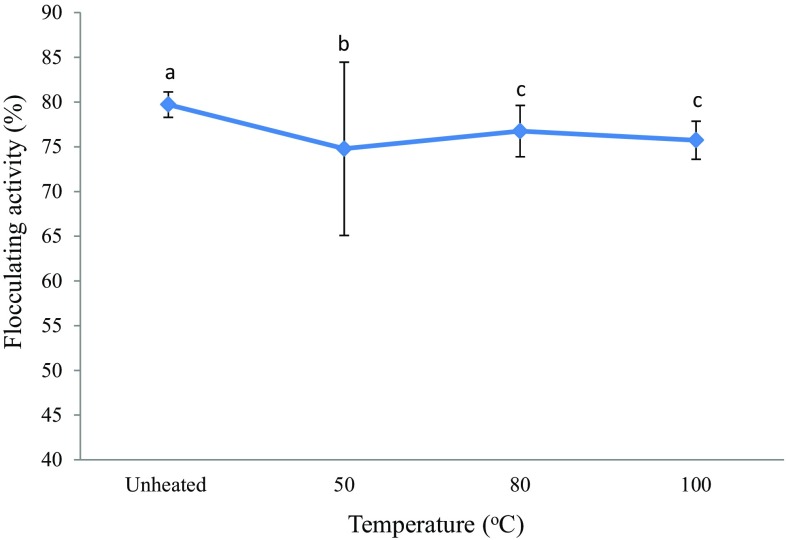
Thermal stability on flocculating activity of the purified bioflocculant. Percentage of flocculating activity with *different letters* (*a*, *b*, and *c*) are significantly (*p* < 0.05) different

The relationship between temperature and flocculating efficiency of the purified bioflocculant was examined at a temperature range of 50–100 °C for 1 h as depicted in Fig. 8. The bioflocculant retained more than 74% flocculating rate when subjected to heating at 100 °C for 1 h. Therefore, it was deduced that the bioflocculant was thermostable and its flocculating activity was not affected when the temperature was elevated. Salehizadeh and Shojaosadati (2001) reported that the presence of protein or peptide in the structure of a bioflocculant is generally linked to its sensitivity to heat and those consisting of sugars are more heat-resistant; hence, it can be concluded that the bioflocculant produced by *Bacillus* sp. consisted predominantly of polysaccharide. The thermal stability of this bioflocculant may be due to the presence of a hydroxyl group that is responsible in the formation of hydrogen bonds in its structure (Ugbenyen and Okoh 2014). Cosa and Okoh (2014) reported a residual flocculating activity of more than 80% for a purified bioflocculant produced by the consortium of *Halobacillus* sp. Mvuyo and *Oceanobacillus* sp. Pinky after heating at 100 °C for 1 h, thus indicating its thermo-stability.

#### Effect of pH on the flocculating activity of purified bioflocculant

**Fig. 9:**
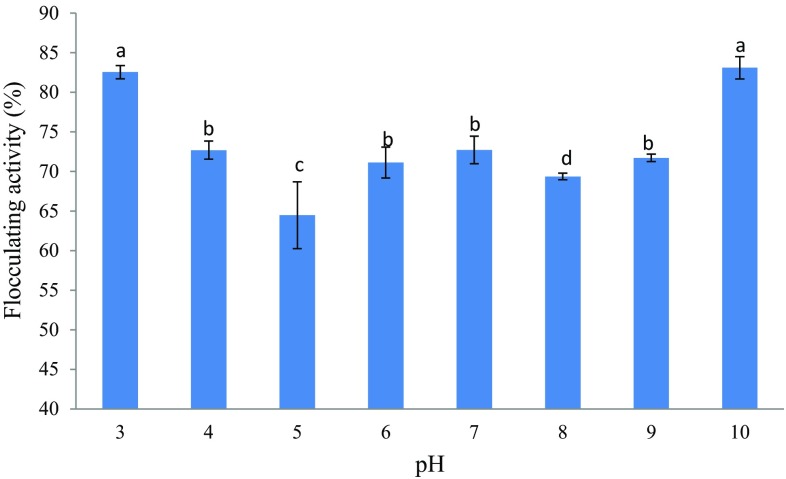
Effect of pH on flocculating activity of the purified bioflocculant. Percentage of flocculating activity with *different letters* (*a*, *b*, *c* and *d*) are significantly (*p* < 0.05) different

The pH has a great impact on flocculating activity (Cosa and Okoh 2014). The flocculating activity of the purified bioflocculant was tested within the pH range of 3–10 as illustrated in Fig. 9. A strong flocculating activity was observed over a wide range of pH 3–10. Maximum flocculating activity was achieved at very acidic pH 3 (83%) and basic pH 10 (83%). This may be due to the bioflocculant exhibiting different electric states at different pH; hence, affecting the bridging efficiency of the bioflocculant for clay powder (Yong et al. 2009). A sharp decline in flocculating activity was observed at pH 5; this might be due to the arrangement of the surface charge which is both pH and temperature dependent. Hence, it can be deduced that the spatial charge arrangements for flocculation were not ambient at pH 5 (Okaiyeto et al. 2015). This bioflocculant showed excellent flocculating activity in both strong acidic and basic conditions. This studied bioflocculant flocculated at a wide pH range and this suggests that its industrial applicability to treat various waters or wastewaters without having to adjust the pH of the water thus rendering the bioflocculant cost-effective (Okaiyeto et al. 2015). Similarly, Zaki et al. (2012) also reported similar results, whereby a bioflocculant produced by *Bacillus velezensis* 404 was stable at the pH range of 3–9 and reached its maximum stability at pH 7. Different microorganisms have been reported to produce bioflocculants with optimal activity at varying pH values (Wang et al. 2011). Liu et al. (2009) reported on a bioflocculant extracted from sludge, named as M-1 as showing high flocculating activity over 74% in the wider pH range of 3–11, whereby the maximum flocculating activity (93%) was achieved at pH 5. It was stated in the literature that at low pH both the bioflocculant and the Kaolin particles are likely to absorb the hydrogen ions (H^+^), consequently weakening the bioflocculant–Kaolin complex mediated by Ca^2+^ and a similar effect was also observed at high pH values due to OH− ions (He et al. 2010).

## Conclusions

This study demonstrated the potential of *Bacillus* sp. isolated from water samples of Thyume River for bioflocculant production when starch, yeast extract and Ca^2+^ were used as sole carbon, nitrogen and cation sources, respectively. The bioflocculant showed outstanding flocculating activity in both strong acidic and basic conditions. The production of the bioflocculant produced by *Bacillus* sp. was found to be associated with cell growth. The FTIR spectrum showed the presence of carboxyl, hydroxyl and methoxyl groups in its molecular chain which are important for flocculation. This bioflocculant consists mainly of polysaccharide which is elucidated by its thermo-stability over a wide range of temperature; a characteristic feature portends its industrial applicability and further studies on practical applications for large-scale production of the bioflocculant is part of on-going research in our laboratory.
